# Epigallocatechin-3-gallate Ameliorates Seawater Aspiration-Induced Acute Lung Injury via Regulating Inflammatory Cytokines and Inhibiting JAK/STAT1 Pathway in Rats

**DOI:** 10.1155/2014/612593

**Published:** 2014-02-20

**Authors:** Wei Liu, Mingqing Dong, Liyan Bo, Congcong Li, Qingqing Liu, Yanyan Li, Lijie Ma, Yonghong Xie, Enqing Fu, Deguang Mu, Lei Pan, Faguang Jin, Zhichao Li

**Affiliations:** ^1^Department of Pulmonary Diseases, Tangdu Hospital, Fourth Military Medical University, Xi'an 710038, China; ^2^Department of Pathology and Pathophysiology, Fourth Military Medical University, Xi'an 710032, China

## Abstract

Signal transducers and activators of transcriptions 1 (STAT1) play an important role in the inflammation process of acute lung injury (ALI). Epigallocatechin-3-gallate (EGCG) exhibits a specific and strong anti-STAT1 activity. Therefore, our study is to explore whether EGCG pretreatment can ameliorate seawater aspiration-induced ALI and its possible mechanisms. We detected the arterial partial pressure of oxygen, lung wet/dry weight ratios, protein content in bronchoalveolar lavage fluid, and the histopathologic and ultrastructure staining of the lung. The levels of IL-1, TNF-**α**, and IL-10 and the total and the phosphorylated protein level of STAT1, JAK1, and JAK2 were assessed in vitro and in vivo. The results showed that EGCG pretreatment significantly improved hypoxemia and histopathologic changes, alleviated pulmonary edema and lung vascular leak, reduced the production of TNF-**α** and IL-1, and increased the production of IL-10 in seawater aspiration-induced ALI rats. EGCG also prevented the seawater aspiration-induced increase of TNF-**α** and IL-1 and decrease of IL-10 in NR8383 cell line. Moreover, EGCG pretreatment reduced the total and the phosphorylated protein level of STAT1 *in vivo* and *in vitro* and reduced the phosphorylated protein level of JAK1 and JAK2. The present study demonstrates that EGCG ameliorates seawater aspiration-induced ALI via regulating inflammatory cytokines and inhibiting JAK/STAT1 pathway in rats.

## 1. Introduction

Seawater drowning is an accident that usually occurs in navigation, operation on sea, and naval warfare. Seawater aspiration-induced acute lung injury with a rapid progress and a high mortality rate is a common complication of seawater drowning [1]. Similar to stress situations such as trauma, burns, and sepsis, seawater aspiration-induced acute lung injury is also characterized by an acute inflammatory process in pulmonary parenchyma and interstitial tissue, severe hypoxemia, and pulmonary edema [2].

Epigallocatechin-3-gallate (EGCG) is the major catechin in green tea that is an extremely popular beverage worldwide. Several previous studies have shown that EGCG had beneficial effects on inflammation, inhibited chemokine-induced neutrophil chemotaxis *in vitro*, and attenuated LPS-induced lung injury [3, 4]. Inflammation plays a critical role in acute lung injury (ALI), whereas JAK/STAT1 pathway promotes inflammation in ALI. EGCG has been reported to reduce STAT1 phosphorylation in various human cells [5]. Therefore, we hypothesized that EGCG might also protect seawater aspiration-induced ALI. In our study, we firstly demonstrated that EGCG pretreatment attenuated seawater aspiration-induced ALI via regulating inflammatory cytokines and inhibiting JAK/STAT1 pathway. These findings suggested that EGCG might have a potential application to prevent and treat the seawater aspiration-induced ALI.

## 2. Materials and Methods

### 2.1. Animals and Materials

**Figure 1:**
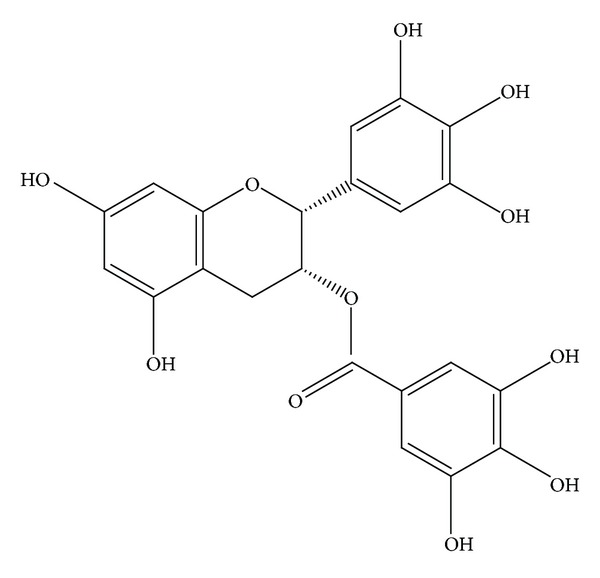
Structures of epigallocatechin-3-gallate (EGCG).

Adult male SD rats (180–220 g) were obtained from the Animal Center of the Fourth Military Medical University (Xi'an, China). The rats were housed in air-filtered, temperature-controlled units with free access to food and water. The experiments were approved by the Animal Care and Use Committee of the Fourth Military Medical University and in accordance with the Declaration of the National Institutes of Health Guide for Care and Use of Laboratory Animals (publication No. 85-23, revised 1985). EGCG (purity > 99%) (Figure 1) was purchased from Sigma Chemical Company (St. Louis, MO, USA), prepared as stock solution in normal saline. Seawater (osmolality 1300 mmol/L, pH 8.2, specific weight 1.05, NaCl 26.518 g/L, MgSO_4_ 3.305 g/L, MgCl_2_ 2.447 g/L, CaCl_2_ 1.141 g/L, KCl 0.725 g/L, NaHCO_3_ 0.202 g/L, and NaBr 0.083 g/L) was prepared according to the major composition of the East China Sea provided by Chinese Ocean Bureau. Anti-STAT1 and anti-phospho-STAT1-Tyr-701 polyclonal antibodies were purchased from Cell Signalling Technology (CST, USA). Anti-JAK1, anti-phospho-JAK1 (PY1022/1023), anti-JAK2, and anti-phospho-JAK2 (PY1007/1008) polyclonal antibodies were purchased from Epitomics Biotechnology Company (EPI, USA). Anti-*β*-actin monoclonal antibody was purchased from Santa Cruz Biotechnology Inc. (Santa Cruz, CA, USA). Enzyme-linked immunosorbent assay (ELISA) kits of IL-1, TNF-*α*, and IL-10 were purchased from R&D Systems (Minneapolis, MN, USA).

### 2.2. Animal Model and Grouping

The seawater aspiration model was produced according to the method described in previous reports with some modifications. The rats were anesthetized with 3% pentobarbital sodium (1.5 mL/kg of body wt, administered i.p.) before trachea was exposed.

SD rats were randomly assigned to four groups (all *n* = 12): control group, EGCG group, seawater group, and seawater + EGCG group. Rats received intraperitoneal injection of 1 mL normal saline with (for EGCG and seawater + EGCG group) or without (for control and seawater group) EGCG (10 mg/kg) 0.5 h before trachea was exposed. In seawater and seawater + EGCG group, seawater (4 mL/kg) was instilled into rat lungs through trachea in 4 min at a steady speed with a 2 mL syringe. The rats were sacrificed by aortic transection at 6 h after trachea was exposed. The dosage of EGCG administration was applied on the basis of previous study [4].

### 2.3. PaO_2_ Measurement

After rats were anesthetized, a catheter was inserted into the right femoral artery to take blood samples. The rats were maintained in the supine position during the first 3 hours of the experiments with the head elevated 30 degrees. Arterial partial pressure of oxygen was measured with a blood gas analyzer. Arterial partial pressure of oxygen in different groups was performed before and 0.5 h, 1 h, 2 h, and 3 h after seawater instillation.

### 2.4. Lung Wet/Dry Ratios

To quantify the magnitude of pulmonary edema, we evaluated the wet/dry weight ratios of lung samples, which were from the upper and middle lobes of right lung in each rat. Tissue samples were obtained 6 h after seawater instillation and weighed immediately after removal, and then they were subjected to desiccation in an oven at 70°C until a stable dry weight was achieved after 72 h. The wet to dry weight ratios were then calculated to quantify the magnitude of pulmonary edema.

### 2.5. Preparation of BALF and Measurements

Rats were anesthetized 6 h after seawater instillation. The left lungs were lavaged with 1 mL ice-cold phosphate buffered saline five times in all groups. 90% of the bronchoalveolar lavage fluid (BALF) was got. The collected BALF was centrifuged at 520 g for 20 min at 4°C and the supernatant was frozen at −80°C for subsequent protein study. Protein concentration in BALF was measured by Lowry's method with bovine serum albumin as a standard.

### 2.6. Lung Histology and Immunohistochemistry

At the end of the experiments, the lung tissues in all groups were removed and fixed with 4% paraformaldehyde for 24 h. The tissues were embedded in paraffin and cut into 5 um sections. Hematoxylin-eosin stains were performed using standard protocol. For immunostainings, sections were deparaffinized, rehydrated in graded alcohols, and blocked by incubating in 0.3% H_2_O_2_ for 30 min. Antigen retrieval was performed by treating the slides in citrate buffer in a microwave oven for 10 min. The slides were incubated for 1 h with normal goat serum and then incubated in a moist chamber with anti-STAT1 and anti-p-STAT1 polyclonal antibody at 4°C overnight. After a complete wash in phosphate buffered saline (PBS), the tissues were incubated in biotin-labeled goat anti-mouse antibody for 30 min at 37°C, rinsed with PBS, and incubated with avidin-biotin peroxidase complex for 30 min at 37°C. The signal was detected using diaminobenzidine (DAB).

### 2.7. Quantification of Histology and Immunohistochemistry

Five samples were used for the quantification of lung histology in every group. Every animal sample was cut into two slices so that the total number of slices was 10 in every group.

For the quantification of lung histology, the sections were stained (hematoxylin and eosin) and examined and analysed independently by two pathologists who were blind to which group the animals belonged. After viewing approximately 10 fields per sector under low and high power, each section was assigned lung injury score ranging from 0 to 4 based on edema, neutrophil infiltration, hemorrhage, bronchiole epithelial desquamation, and hyaline membrane formation according to previous standard assessment [6]. A score scaled from 0 to 4 represents the severity: 0 for no injury (a normal appearing lung), 1 for modest injury (limited congestion and interstitial edema but no interstitial neutrophilic infiltrate with occasional red blood cells and neutrophils in the alveolar spaces), 2 for intermediate injury (mild congestion, interstitial edema, and interstitial neutrophilic infiltrate with occasional red blood cells and neutrophils in the alveolar spaces), 3 for widespread injury (more prominent congestion and interstitial edema with neutrophils partially filling the alveolar spaces but with consolidation), and 4 for widespread injury (most prominent, marked congestion and interstitial edema with neutrophilic infiltrate nearly filling the alveolar spaces or with frank lung consolidation).

For the quantification of immunohistochemistry, all sections were viewed 10 fields per sector and analyzed quantitatively with an image analysis program (Image Pro Plus version 6.0). Positive brown staining was quantitated as the integrated optical density (IOD) for arbitrary areas. Data were pooled to show a mean value, and a statistical analysis was applied to compare the results obtained from different experimental groups.

### 2.8. Lung Ultrastructure

Electron microscopy was performed on glutaraldehyde fixed lungs from all groups. Lung tissues were cut into 1-2 mm^3^ cubes, rinsed in 0.1 M phosphate buffer (pH = 7.4), postfixed for 1 h in 1% osmium tetroxide, dehydrated in alcohol, and embedded in epoxy resin. Semithin (1 mm thick) sections were stained with 1% toluidine blue to locate interesting areas for electron microscopic examination. Ultrathin sections that stained with uranyl acetate and lead citrate were examined under TEM-100CX electron microscope (Japan Electron Optical Laboratory, Tokyo, Japan).

### 2.9. Cell Culture and Treatment

The rat alveolar macrophage cell line NR8383 (obtained from ATCC, Rockville, MD, USA) was maintained in Ham's F12 medium supplemented with 15% fetal calf serum, 100 U/mL of penicillin, and 100 ug/mL of streptomycin at 37°C in a humidified atmosphere containing 5% CO_2_ and 95% air. After being incubated in the presence or absence of EGCG (10 uM), seawater (30%, 0.3 mL seawater per 1 mL total volume) was added to stimulate the cell for 6 h.

### 2.10. ELISA

Levels of TNF-*α*, IL-1, and IL-10 in the lung homogenate and cells supernatant were determined by using commercially available ELISA kits according to the manufacturer's instructions.

### 2.11. Quantitative Real-Time PCR

Total RNA was isolated and real-time PCR was performed as previously study [7]. The primers for STAT1 (target fragment size 244 bp) were (forward) 5′-CGGGAAGGGGCCATCACATT-3′ and (reverse) 5′-CTGGTGCTTCCTTTGGCCTG-3′, the primers for JAK1 (target fragment size 455 bp) were (forward) 5′-CATCAGGGCCGCACAGGAAT-3′ and (reverse) 5′-TGGGAAGCTCTGGCAACTGC-3′, the primers for JAK2 (target fragment size 532 bp) were (forward) 5′-GAGTGTGGAGATGTGCCGCT-3′ and (reverse) 5′-CCACACATCTGAGGCCACAG-3′, and for *β*-actin (target fragment size 266 bp) they were (forward) 5′-GTCCCTCACCCTCCCAAAAG-3′ and (reverse) 5′-GCTGCCTCAACACCTCAACCC-3′, respectively.

### 2.12. Western Blot

The lungs were perfused with pH 7.4 PBS to remove the blood cells from the pulmonary circulation, and then the tissue samples from each group were collected. Total protein extracted from whole-cell and lung tissues was, respectively, prepared according to instructions of Total Protein Extraction Kit. Protein concentrations were determined by BCA protein assay kit. Samples were separated on a denaturing 8% SDS-polyacrylamide gel and transferred to a nitrocellulose membrane followed by incubation with mouse or rabbit monoclonal antibodies against STAT1 (1 : 500), p-STAT1 (1 : 500), JAK1 (1 : 1000), p-JAK1 (1 : 1000), JAK2 (1 : 1000), p-JAK2 (1 : 1000), and *β*-actin (1 : 1000) overnight. The secondary antibody (anti-mouse or anti-rabbit, 1 : 5000) was incubated. Detection was performed using the enhanced chemiluminescence system (Amersham, Arlington Heights, IL, USA).

### 2.13. Statistical Analysis

Data are expressed as mean ± SEM and statistical analysis was performed with one-way ANOVA, followed by Dunnett's test for multiple comparisons. For noncontinuous histological injury score data, the Kruskal-Wallis test was used. A statistical difference was accepted if *P* < 0.05.

## 3. Results

### 3.1. Effect of EGCG Pretreatment on PaO_2_ in Seawater Aspiration-Induced ALI

**Figure 2:**
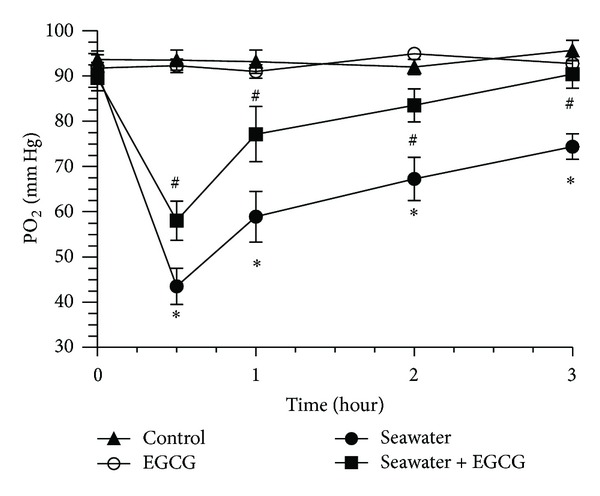
Effect of EGCG on PaO_2_ in seawater aspiration-induced ALI. Seawater aspiration-induced ALI caused a significant decrease of PaO_2_ in a time dependent manner, which could be partly reversed by EGCG pretreatment. Data were presented as means ± SEM (*n* = 8). *P < 0.05 versus control group; ^#^ *P* < 0.05 versus seawater group.

As shown in Figure 2, seawater aspiration caused a significant decrease in PaO_2_ in a time dependent manner. The most serious hypoxemia occurred at the time point of 0.5 h after seawater instillation. However, EGCG pretreatment partly reversed the seawater aspiration-induced decrease of PaO_2_. In seawater + EGCG group, PaO_2_ almost reached the normal level at the time point of 3 h after seawater instillation.

### 3.2. Effect of EGCG Pretreatment on the Lung Edema in Seawater Aspiration-Induced ALI

**Figure 3:**
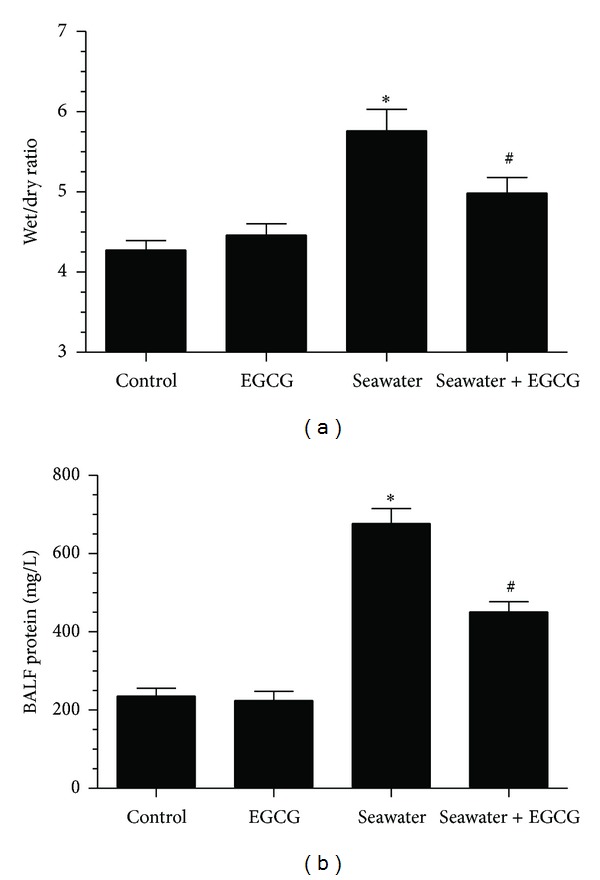
Effect of EGCG pretreatment on the lung edema (a) and the concentrations of total protein in BALF (b). EGCG significantly decreased lung wet/dry ratios and total protein in BALF in seawater aspiration-induced ALI rats. Data were presented as mean ± SEM (*n* = 12 for lung wet/dry ratios and *n* = 6 for total protein in BALF). *P < 0.05 versus control group; ^#^ *P* < 0.05 versus seawater group.

To investigate the effect of EGCG on lung edema, we measured the lung wet/dry ratios (Figure 3(a)). The results showed that seawater aspiration significantly increased the lung wet/dry ratios compared with the control group (from 4.27 ± 0.12 to 5.76 ± 0.23, *P* < 0.05, *n* = 12), and EGCG pretreatment markedly reduced the lung wet/dry ratios (from 5.76 ± 0.23 to 4.98 ± 0.20, *P* < 0.05, *n* = 12). At the same time, there were no significant differences between the control and EGCG groups, which indicated that EGCG did not affect lung water content in rats.

### 3.3. Effect of EGCG Pretreatment on the Concentrations of Total Protein in BALF in Seawater Aspiration-Induced ALI

The concentrations of total protein in BALF increased after seawater instillation compared with the control group (from 235.75 ± 20.77 to 676.75 ± 38.39, *P* < 0.05, *n* = 6). EGCG pretreatment can obviously decrease the level of protein in BALF of seawater aspiration-induced acute lung injury (from 676.75 ± 38.39 to 451.00 ± 26.12, *P* < 0.05, *n* = 6) (Figure 3(b)).

### 3.4. Effect of EGCG Pretreatment on Histopathologic Changes and the Lung Injury Scores in Seawater Aspiration-Induced ALI

**Figure 4:**
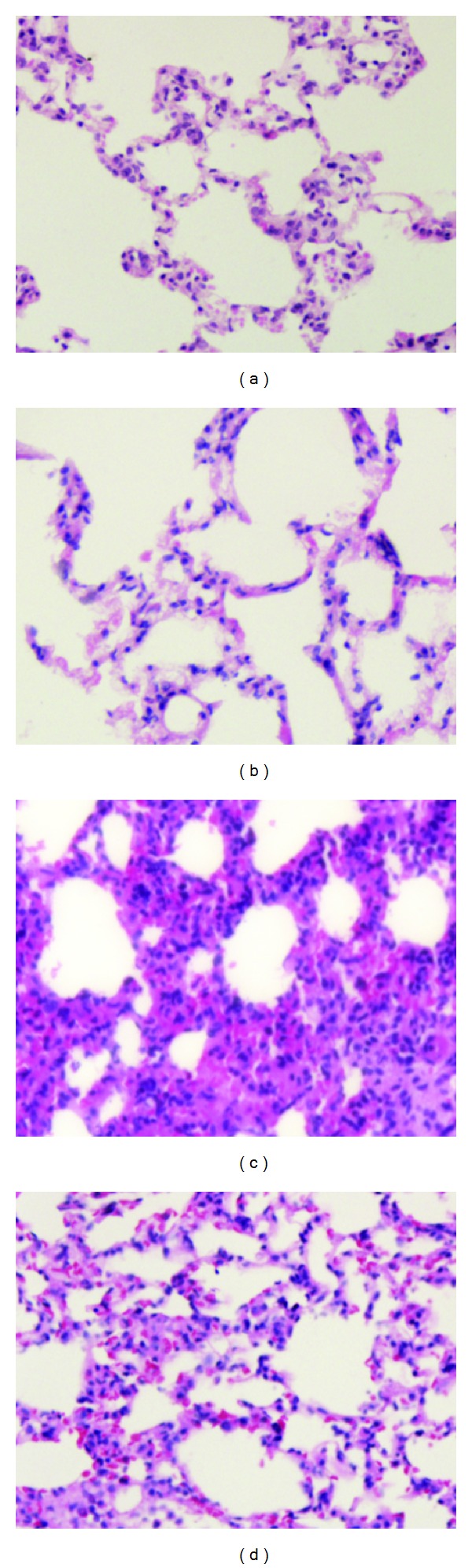
Effect of EGCG pretreatment on the histopathologic changes in lungs in seawater aspiration-induced ALI: (a) control group: the lung structure was normal; (b) EGCG (10 mg/kg) group: there was no obvious change in lung structure compared with control group; (c) seawater group (4 mL/kg): alveolar wall thickened, edema and hemorrhage, less alveolar space and obvious inflammatory cells infiltration into interstitial and alveolar spaced; (d) seawater (4 mL/kg) + EGCG (10 mg/kg): lung injury was significantly alleviated compared with seawater group (hematoxylin and eosin staining, original magnification 200×).

Histopathologic staining showed that seawater aspiration induced pulmonary edema, infiltration of inflammatory cells in the lung tissues and alveoli, hemorrhage, bronchiole epithelia desquamation, and alveolar collapse compared with the control group (Figure 4(c)). EGCG pretreatment improved these seawater aspiration-induced histopathologic changes compared with the seawater group (Figure 4(d)). There was no obvious change in lung structure in the control and EGCG groups (Figures 4(a) and 4(b)). The assigned lung injury scores of every group for edema, infiltration of neutrophil, hemorrhage, bronchiole epithelia desquamation and hyaline membrane were shown in Table 1.

### 3.5. Effect of EGCG Pretreatment on the Expression of STAT1 and p-STAT1 in Lungs in Seawater Aspiration-Induced ALI in Rats

**Figure 5:**
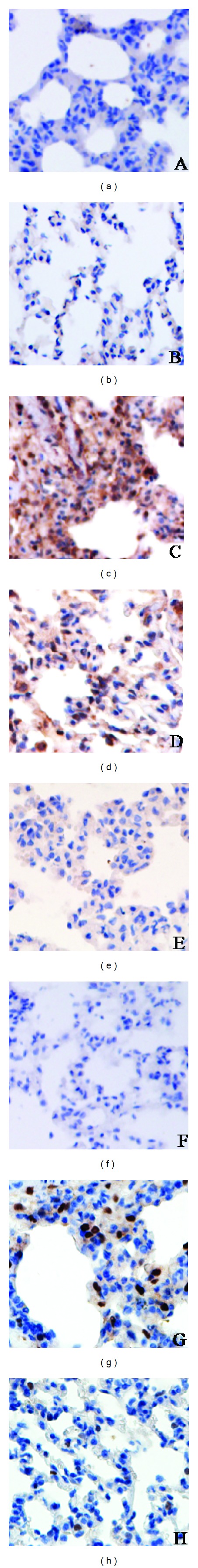
Effect of EGCG pretreatment on the expression of STAT1 and p-STAT1 in lungs in seawater aspiration-induced ALI: (a)–(d) the expression of STAT1; (e)–(h) the expression of p-STAT1; (a) and (e) the control group; (b) and (f) the EGCG group; (c) and (g) the seawater group; (d) and (h) the seawater + EGCG group (original magnification 200×).

The immunohistochemistry staining showed that the expression of STAT1 and p-STAT1 significantly increased after seawater aspiration (Figures 5(a), 5(c), 5(e), and 5(g)). EGCG pretreatment inhibited the increase of STAT1 and p-STAT1 (Figures 5(c), 5(d), 5(g), and 5(h)), whereas there was no difference of STAT1 and p-STAT1 between the control and EGCG group (Figures 5(a), 5(b), 5(e), and 5(f)). The corresponding IOD values of STAT1 and p-STAT1 immunohistochemistry staining for every group are shown in Table 2.

**Figure 6:**
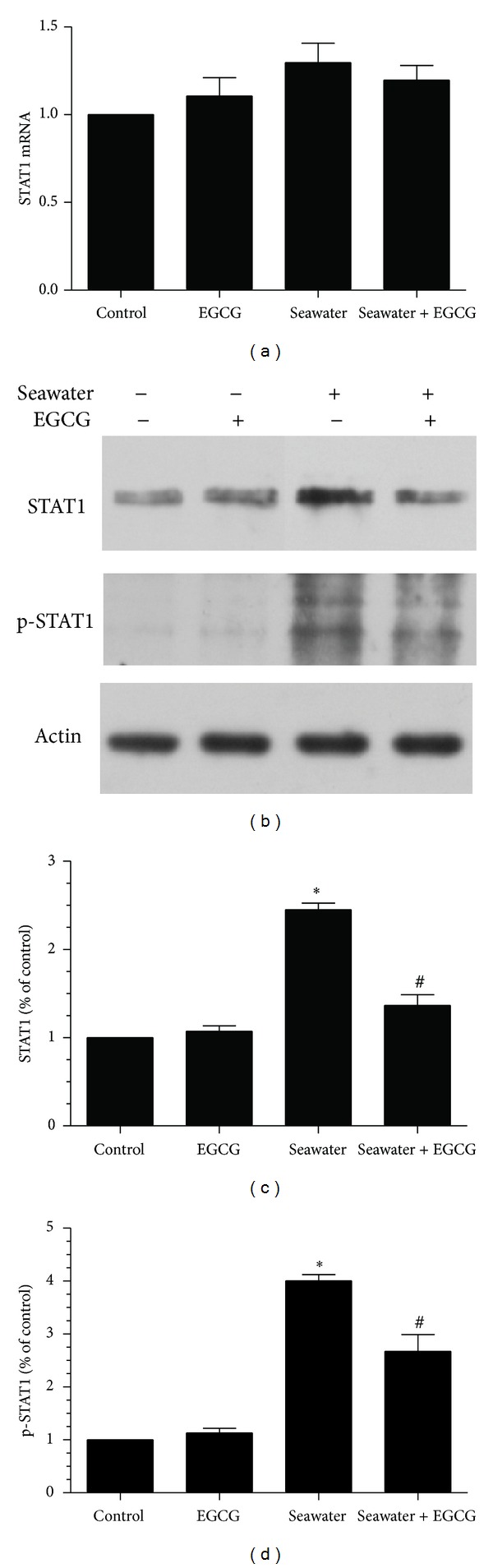
Effects of EGCG pretreatment on the expression of STAT1 and p-STAT1 in rats. (a) mRNA expression of STAT1 in rats; (b) representative western blot for the phosphorylated STAT1 and the total STAT1 protein in different groups. Samples were prepared for western blot analysis using anti-STAT1, anti-p-STAT1, and anti-*β*-actin; (c)-(d) summarized data of the total (c) and the phosphorylated (d) STAT1 protein in different groups. Data represent three independent experiments and are expressed as mean ± SEM; *P < 0.05 versus control group; ^#^ *P* < 0.05 versus seawater group.

Further, we measured the mRNA level of STAT1 and the protein levels of STAT1 and p-STAT1 in lungs using qPCR and western blot. The mRNA expression of STAT1 was not significantly different between groups (Figure 6(a)). However, seawater aspiration increased both the total and the phosphorylated protein levels of STAT1 (Figure 6(c)). EGCG remarkably inhibited the seawater aspiration-induced increase of both STAT1 and p-STAT1 (Figure 6(d)).

### 3.6. Effect of EGCG Pretreatment on the Pulmonary Ultrastructure in Seawater Aspiration-Induced ALI

**Figure 7:**
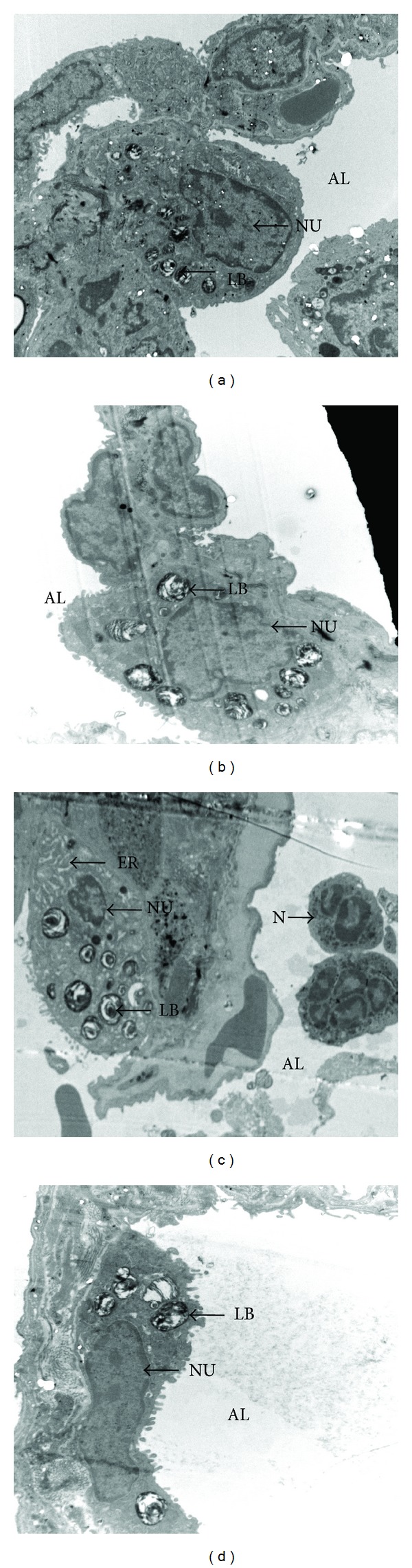
Effect of EGCG pretreatment on pulmonary ultrastructure. (a) Control group: the normal alveolar epithelial cell type II and thin alveolar (AL) wall. (b) EGCG group: there was no obvious change in pulmonary ultrastructure compared with control group. (c) Seawater group: neutrophil (N) infiltration in the alveolar lumen and thickened alveolar wall were observed. Meanwhile, endoplasmic reticulum expanded and lamellar bodies (LB) emptying increased. (d) Seawater + EGCG group: the change of alveolar wall and the alveolar epithelial cell type II were alleviated compared with seawater group ((a) 5000×; (b) 4000×; (c) 5000×; (d) 8000×). NU: nucleus; ER: endoplasmic reticulum.

Lungs from the control group showed normal ultrastructure (Figure 7(a)). Lungs from the seawater group showed diffuse thickening of the alveolar wall and neutrophil infiltration in the alveolar lumen and increased number of lamellar bodies. Meanwhile, endoplasmic reticulum expanded and lamellar bodies emptying increased (Figure 7(c)). EGCG pretreatment alleviated neutrophil infiltration, the alveolar wall thickening, and the change of the alveolar epithelial cell type II in seawater aspiration-induced ALI (Figure 7(d)). There was no obvious change in pulmonary ultrastructure compared with the control group (Figure 7(b)).

### 3.7. Effect of EGCG Pretreatment on TNF-*α*, IL-1, and IL-10 Levels in Seawater Aspiration-Induced ALI

**Figure 8:**
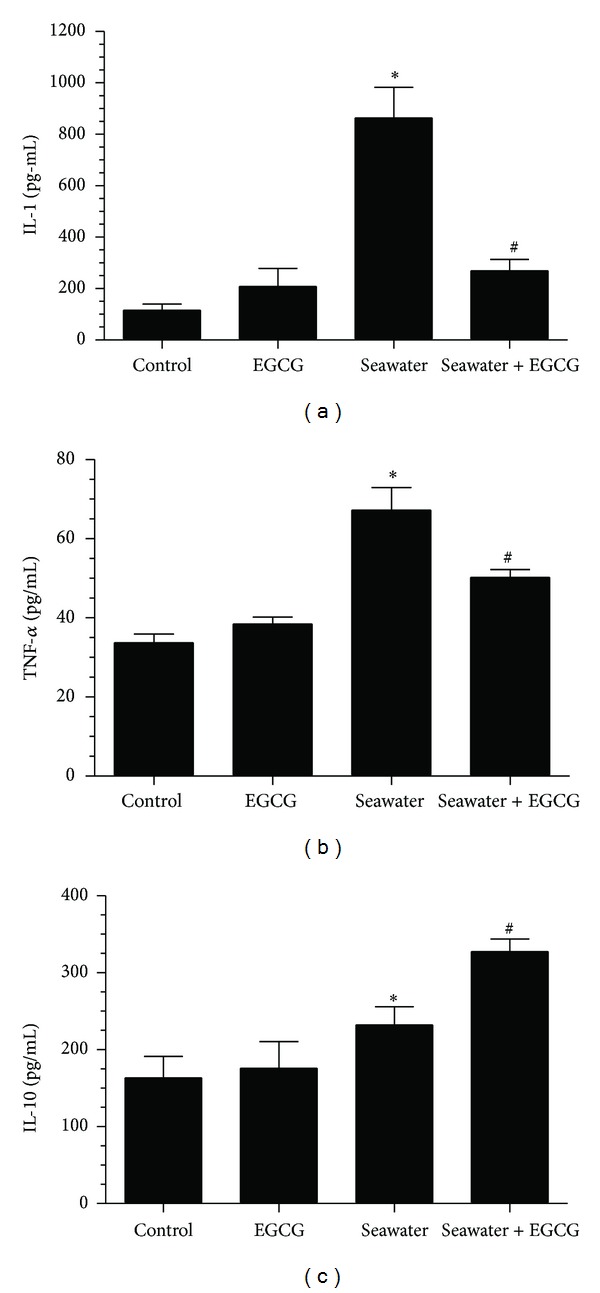
Effects of EGCG pretreatment on IL-1, TNF-*α*, and IL-10 of lung tissue in seawater aspiration-induced ALI. IL-1 (a), TNF-*α* (b), and IL-10 (c) increased in seawater group. EGCG pretreatment downregulated the levels of IL-1 and TNF-*α* and upregulated the level of IL-10. Data were presented as mean ± SEM (*n* = 12). *P < 0.05 versus control group; ^#^ *P* < 0.05 versus seawater group.

**Figure 9:**
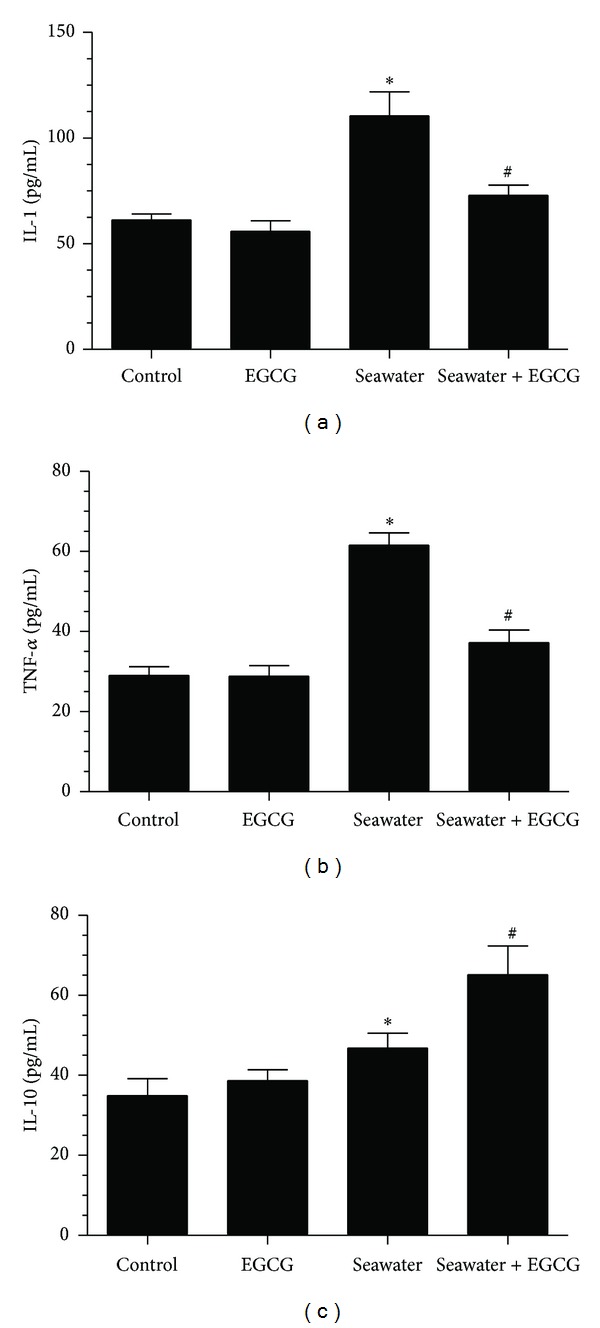
Effects of EGCG pretreatment on IL-1, TNF-*α*, and IL-10 in NR8383 cells. IL-1 (a), TNF-*α* (b), and IL-10 (c) increased in seawater group. EGCG pretreatment decreased IL-1 and TNF-*α* but increased IL-10. Data were presented as mean ± SEM (*n* = 12). *P < 0.05 versus control group; ^#^ *P* < 0.05 versus seawater group.

TNF-*α*, IL-1, and IL-10 in lungs increased after seawater instillation (from 33.69 ± 2.19 to 67.21 ± 5.76 for TNF-*α*, from 119.52 ± 23.67 to 863.64 ± 119.25 for IL-1, and from 163.51 ± 27.53 to 232.27 ± 23.53 for IL-10, all *P* < 0.05, *n* = 12), whereas EGCG pretreatment inhibited the increase of TNF-*α* and IL-1 and promoted the increase of IL-10 (from 67.21 ± 5.76 to 50.25 ± 1.95 for TNF-*α*, from 863.64 ± 119.25 to 269.59 ± 44.31 for IL-1, and from 232.27 ± 23.53 to 327.41 ± 16.48 for IL-10, all *P* < 0.05, *n* = 12) (Figure 8). Meanwhile, there was no difference between the control and EGCG groups. The same results were obtained in the cell line NR8383 (Figure 9).

### 3.8. Effect of Seawater and EGCG Pretreatment on the Expression of STAT1 and P-STAT1 in NR8383 Cells

**Figure 10:**
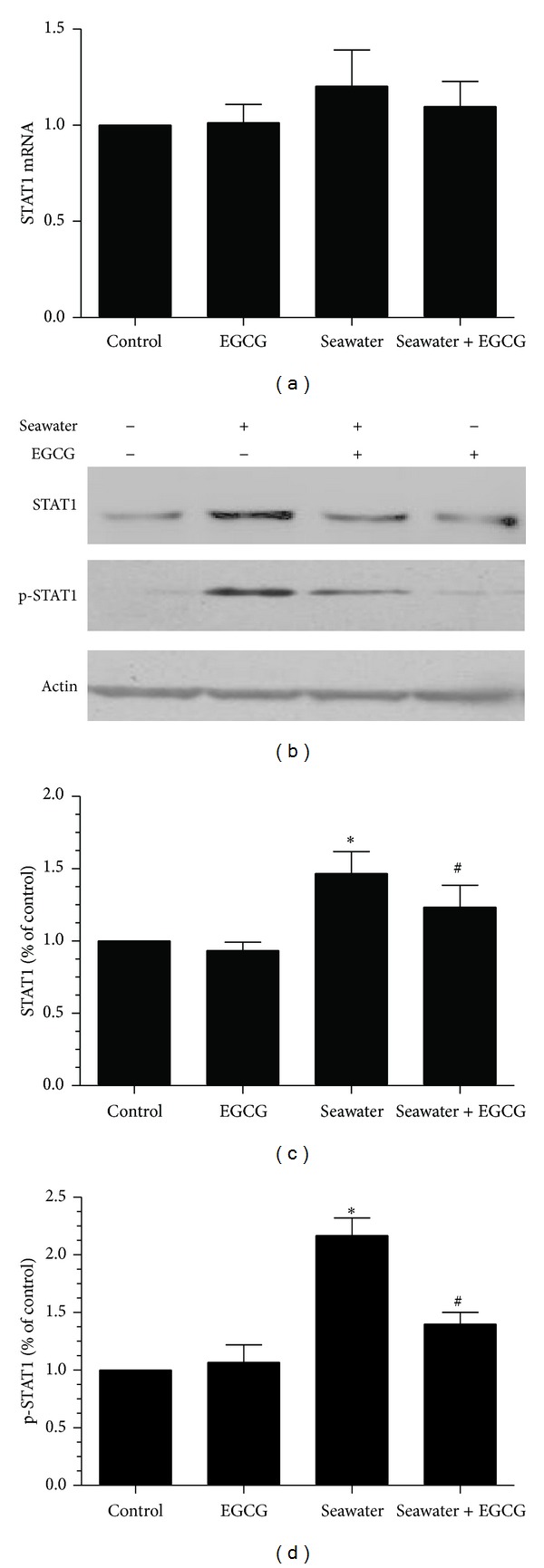
Effect of EGCG pretreatment on the expression of STAT1 and p-STAT1 in the cell line NR8383. (a) mRNA expression of STAT1 in NR8383 cells. (b) Representative western blot for the phosphorylated STAT1 and the total STAT1 protein in different groups. (c)-(d) Summarized data of the total (c) and the phosphorylated (d) STAT1 protein level in different groups. Data represent three independent experiments and are expressed as mean ± SEM; *P < 0.05 versus control group; ^#^ *P* < 0.05 versus seawater group.

Then we further measured the effects of seawater and EGCG pretreatment on the mRNA level of STAT1 and the protein levels of STAT1 and p-STAT1 in NR8383 cell line. The mRNA expression of STAT1 was not significantly different between groups (Figure 10(a)). However, the protein expression of STAT1 and p-STAT1 significantly increased in the seawater group. Pretreated with EGCG efficiently inhibited the increase of STAT1 and P-STAT1 (Figures 10(b)–10(d)).

### 3.9. Effect of Seawater and EGCG Pretreatment on the Expression of JAK1 and JAK2 in NR8383 Cell Line

**Figure 11:**
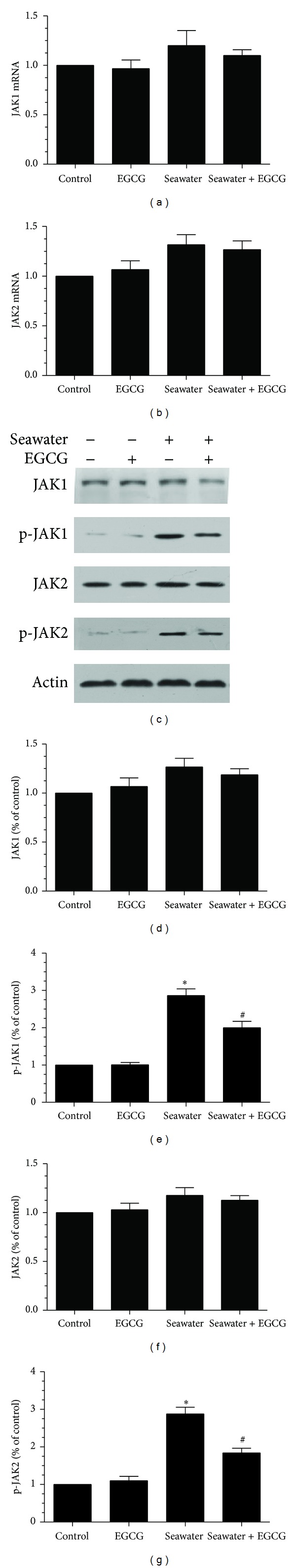
Effect of seawater and EGCG pretreatment on the expression of JAK1, p-JAK1, JAK2 and p-JAK2 in the cell line NR8383. (a) mRNA expression of JAK1 in NR8383 cells. (b) mRNA expression of JAK2 in NR8383 cells. (c) Representative western blot for the JAK1, p-JAK1, JAK2, and p-JAK2 protein in different groups. (d)–(g) Summarized data of the JAK1 (d), p-JAK1 (e), JAK2 (f), and p-JAK2 (g) protein level in different groups. Data represent three independent experiments and are expressed as mean ± SEM; *P < 0.05 versus control group; ^#^ *P* < 0.05 versus seawater group.

To further investigate the mechanism of EGCG protecting seawater aspiration-induced ALI, we evaluated the mRNA and/or protein levels of JAK1, p-JAK1, JAK2, and p-JAK2, which are the upstream regulation molecular of STAT1, in NR8383 cell line. The mRNA expression of JAK1 and JAK2 in the cell line NR8383 was not significantly different between groups (Figures 11(a)-11(b)). The total protein of JAK1 and JAK2 was also not significantly different between groups. However, the levels of p-JAK1 and p-JAK2 significantly increased in the seawater group. Pretreatment with EGCG efficiently prevented the increase of p-JAK1 and p-JAK2 (Figures 11(c)–11(g)).

## 4. Discussion

In our study, we firstly showed that EGCG pretreatment significantly improved hypoxemia and histopathologic changes, alleviated pulmonary edema and lung vascular leak, reduced the level of TNF-*α* and IL-1, and increased the production of IL-10 in seawater aspiration-induced ALI rats. Moreover, EGCG pretreatment decreased the total and the phosphorylated protein level of STAT1 in lungs *in vivo* and *in vitro* and the phosphorylated protein level of JAK1 and JAK2 *in vitro*. These results demonstrated that EGCG pretreatment ameliorated seawater aspiration-induced acute lung injury in rats. The protection effects of EGCG may be due to regulating the inflammatory cytokines and inhibiting JAK/STAT1 pathway.

Seawater aspiration-induced ALI, which is one of the most serious complications of seawater drowning [8], is one kind of acute lung injuries. Clinical studies indicated that phagocyte infiltration and lung edema are the main characteristics of seawater aspiration-induced ALI [9]. Our previous studies also showed that infiltration of inflammatory cells [10] and permeability of alveolar wall [11] increased in seawater aspiration-induced ALI in rats, and the resveratrol's prodrug 3,5,4′-tri-O-acetylresveratrol exhibited a protective effect on seawater aspiration-induced ALI by inhibiting inflammatory response and suppressing the oxidative stress [12].

Previous studies showed that EGCG exhibited the potent anti-inflammation [13], anticancer [3], and antioxidation [14] effects in some studies. *In vitro*, EGCG could decrease inflammatory factor, such as IL-12, IL-6, and TNF-*α*, and the expression of iNOS and COX-2 proteins in LPS-stimulated murine macrophages and bone marrow-derived macrophages [4, 15, 16]. *In vivo*, EGCG can inhibit the expressions of TLR4, NF-*κ*B, HMGB1, TNF-*α*, and IL-1*β*, and promote the expression of IL-10 in neuropathic pain rats [17]. Furthermore, previous study showed that EGCG attenuated LPS-induced lung injury by suppression of the MIP-2 and TNF-*α* production [4].

However, whether EGCG has a protective effect on seawater aspiration-induced ALI remains unknown. In our study, we found that EGCG pretreatment ameliorated hypoxemia and attenuated infiltration of inflammatory cells and the releasing of inflammatory factors alleviated the alveolar wall thickening and the injury change of the alveolar epithelial cell type II in seawater aspiration-induced ALI rats. Moreover, EGCG pretreatment also reduced the wet/dry weight ratios and the total protein in BALF, which suggested that EGCG ameliorated pulmonary vascular permeability in seawater aspiration-induced ALI.

STAT1 has been identified in various organs [18] as a transcription factor, which is the important part of the cell signal pathway JAK/STAT, and plays a key role in inflammation reaction [19]. STAT1 exists in an inactive form in the cytoplasm and can be activated by its upstream molecule phosphorylated JAK1 and JAK2. The activated STAT1 (phosphorylation STAT1) is transported into nucleus and then promotes the upregulation of proinflammatory factors including TNF-*α*, IL-1 [20, 21]. Phosphorylation of STAT1 is the critical step initiating pulmonary inflammation in LPS, gastric acid aspiration, or acute pancreatitis-induced ALI [22]. Meanwhile, hyperosmotic shock activates STAT1 and leads to STAT1 phosphorylation in diverse cell line and tissues [23–25]. Therefore, the activated JAK1 and JAK2 induced the increase of phosphorylated STAT1 maybe one of the most important mechanisms that cause inflammation and lung tissue collapse in hyperosmotic seawater-induced ALI. Previous studies showed that EGCG exhibited anti-inflammatory action through inhibiting STAT1 activation and downregulating JAK/STAT1 pathway [26, 27]. Our results showed that the phosphorylated STAT1 increased in lungs in seawater aspiration-induced ALI rats; *in vitro* studies showed that the increase of phosphorylated STAT1 is accompanied by the increase of the phosphorylated JAK1 and JAK2. So JAK/STAT pathway maybe plays an important role in the seawater aspiration-induced ALL. In addition, seawater aspiration had no effects on the mRNA level but increased the total protein level of STAT1 in lungs in the animal model (Figures 5 and 6). The increase of the total STAT1 protein may be owing to its phosphorylation by JAK1 and JAK2. Previous study has shown that the JAK1 and JAK2 could phosphorylate STAT1 and inhibit its degradation [26].

In the present study, EGCG markedly attenuated the seawater-induced phosphorylation of JAK1, JAK2, and STAT1 *in vitro*, attenuated the increase of STAT1 and p-STAT1, reduced the proinflammatory cytokines, and increased anti-inflammatory cytokines *in vivo* and vitro. So our results suggest that protection effect of EGCG on seawater aspiration-induced ALI is partly due to its regulation of inflammatory cytokines and inhibition of JAK/STAT1 pathway.

## 5. Conclusions

In conclusion, we firstly demonstrated that EGCG pretreatment attenuated seawater aspiration-induced ALI in rats with a marked reversal of inflammatory events and ameliorative hypoxemia, associated with inhibiting JAK/STAT1 pathway. These findings suggested that EGCG might have a potential application to prevent and treat the seawater aspiration-induced ALI.

## Figures and Tables

**Table 1:** Effects of EGCG on the assigned lung injury scores in seawater aspiration-induced ALI.

Group	Edema	Neutrophil infiltration	Hemorrhage	Bronchiole epithelial desquamation	Hyaline membrane
Control	0.2 ± 0.12	0.3 ± 0.15	0.1 ± 0.24	0.2 ± 0.27	0.0 ± 0.00
EGCG	0.3 ± 0.11	0.2 ± 0.14	0.2 ± 0.31	0.1 ± 0.29	0.0 ± 0.00
Seawater	3.5 ± 0.26*	3.6 ± 0.36*	2.9 ± 0.34*	3.2 ± 0.28*	0.3 ± 0.31
Seawater + EGCG	1.7 ± 0.33^#^	1.6 ± 0.29^#^	1.7 ± 0.27^#^	1.5 ± 0.31^#^	0.2 ± 0.28

Note: data are represented as mean ± SEM; *P < 0.05 versus control group; ^#^ *P* < 0.05 versus seawater group; *n* = 10.

**Table 2:** Comparison of the quantitated IOD of STAT1 and p-STAT1 staining in lung in seawater aspiration-induced ALI.

Groups	IOD of STAT1	IOD of P-STAT1
Control	2.02 ± 0.34	1.53 ± 0.37
EGCG	2.24 ± 0.16	1.75 ± 0.28
Seawater	6.82 ± 0.37*	5.67 ± 0.39*
Seawater + EGCG	4.67 ± 0.34^#^	3.42 ± 0.29^#^

Note: difference in immunohistochemical staining evaluated by image analysis program. Data are represented as mean ± SEM; *P < 0.05 versus control group; ^#^ *P* < 0.05 versus seawater group; *n* = 10.
